# Applying quality improvement tools to the Reaching Every District strategy: a mixed-methods examination of a systems strengthening approach to building the capacity of immunization managers and service providers in Ethiopia

**DOI:** 10.11604/pamj.2023.44.180.35810

**Published:** 2023-04-17

**Authors:** Lisa Oot, Zenaw Adam, Tewodros Alemayehu, Adriana Almiñana, Belayneh Dagnew, Daniel Girma, Elena Herrera, Natasha Kanagat, Quail Rogers-Bloch

**Affiliations:** 1 JSI Research & Training Institute, Inc. (JSI), Arlington, Virginia, United States of America,; 2 JSI Research & Training Institute, Inc. (JSI), Addis Ababa, Ethiopia

**Keywords:** Reaching Every District, quality improvement, routine immunization, vaccination, program strengthening, performance improvement, data quality and use, equity, zero dose

## Abstract

The Reaching Every District (RED) strategy, implemented in Ethiopia for over 15 years, has helped to improve immunization performance. However, recent demographic and health survey data indicate wide variations in immunization coverage. To address these disparities, quality improvement (QI) tools and methods were applied in phases to the RED strategy between 2011 and 2018 and were ultimately scaled to 103 districts in Ethiopia. Quantitative and qualitative data were collected from 2015-2018 to examine RED-QI uptake, practices, sustainability, and effects on Ethiopia´s routine immunization (RI) system. Qualitative interviews examined how RED-QI practices were carried out in each district, and quantitative data from a sample of health facilities provided information on the effects of RED-QI on the RI system. The RED-QI intervention increased the capacity of immunization managers and health workers to plan, implement, and monitor immunization activities, achieving expanded reach and enhancing the quality of services. RED-QI strengthened health workers´ capacity to identify and target communities for immunization, including in hard-to-reach areas. Improved planning resulted in expanded reach and greater equity in services. Immunization staff experienced enhanced capacity to plan immunization services, design approaches to address local challenges, reach target populations, and use data to monitor program performance. While challenges were noted with certain QI tools, assessments indicate that the RED-QI approach can be used in diverse contexts to strengthen RI.

## Introduction

The goal of reaching every child with life-saving vaccinations has been at the heart of immunization programs for decades. However, in 2019, an estimated 14 million children under one year of age did not receive any vaccinations and a further 5.7 million children were only partially vaccinated, leaving them susceptible to vaccine-preventable diseases [1]. Gavi´s strategy for 2021-2025 (i.e., Gavi 5.0) and WHO´s Immunization Agenda 2030 (i.e., IA2030) are ambitious global immunization initiatives that emphasize improving immunization equity, tailoring strategies to reach missed communities, and working to build resilient, integrated primary health care systems [2,3]. Achieving universal immunization requires that routine immunization (RI) systems have strong subnational management to ensure quality services are provided to all intended beneficiaries.

In 2002, the World Health Organization (WHO) introduced the Reaching Every District (RED) strategy, which focused on strengthening district-level immunization service delivery [4,5]. Assessments of the strategy suggest that it effectively improves immunization service delivery. However, findings also suggest that countries struggle to implement all five components of RED, indicating a need to explore approaches that support full implementation [4,6-8]. Quality improvement (QI) tools and methods have been used to strengthen the delivery of health interventions, especially in relation to strengthening skills in local problem solving [9-11]. In Uganda, Bazos *et al*. [12] found that a QI approach grounded in systems thinking and coupled with intensive coaching enhanced the implementation of the RED strategy. A recent study by Manyazewal *et al*. [13] found that the use of continuous QI interventions increased immunization capacity and vaccination coverage in Ethiopia. In this paper, we seek to demonstrate how applying QI processes and tools to the RED strategy improved the capacity of immunization managers and service providers to plan, implement, and monitor equitable immunization services in Ethiopia.

**Status of routine immunization in Ethiopia:** since introducing the RED strategy nationally in 2003, Ethiopia has made impressive gains in improving immunization coverage. However, the implementation of all five RED components across all Ethiopian districts has been difficult to operationalize [14], which may contribute to ongoing disparities in immunization coverage. According to the most recent Ethiopian mini Demographic and Health Surveys (DHS 2019), [15] the immunization coverage rate for a third dose of diphtheria-tetanus-pertussis containing vaccine (DTP3) is 61 percent, up from 21 percent in 2000 [16] (DHS, 2000) before the introduction of RED, yet still falls below the global target of 90 percent. Furthermore, there are wide variations in immunization coverage across geographic locations, income levels, and maternal education. To address coverage disparities, the Ethiopian Ministry of Health (MoH) asked for support in the design and implementation of innovative strategies to address coverage and service quality inequities that could be scaled and sustained within the country.

**The RED-QI intervention:** to address low and inequitable immunization coverage in Ethiopia, JSI Research & Training Institute, Inc. (JSI) applied concepts and tools from the field of QI to help immunization programs at the district, sub-district, and facility-level operationalize RED and put all five of its components into practice [17-19]. This approach is known as Reaching Every District using Quality Improvement (RED-QI) [19-22]. Through this paper, we seek to answer the following questions: 1) Did the application of QI tools to a system strengthening strategy (i.e., RED) improve the capacity of the health system to deliver routine immunization services? 2) Did RED-QI strengthen management and service delivery processes to improve service equity? If yes, how was this done? This paper includes a summary of the multiple assessments and data collected through the larger project of which RED-QI was a part. This paper does not discuss the effects of the RED-QI approach on immunization coverage. Campbell *et al*. [23] describe how the RED-QI approach demonstrated improvements in coverage of the pentavalent vaccine, vaccination timeliness, and a reduction in missed measles vaccinations.

**Figure 1:**
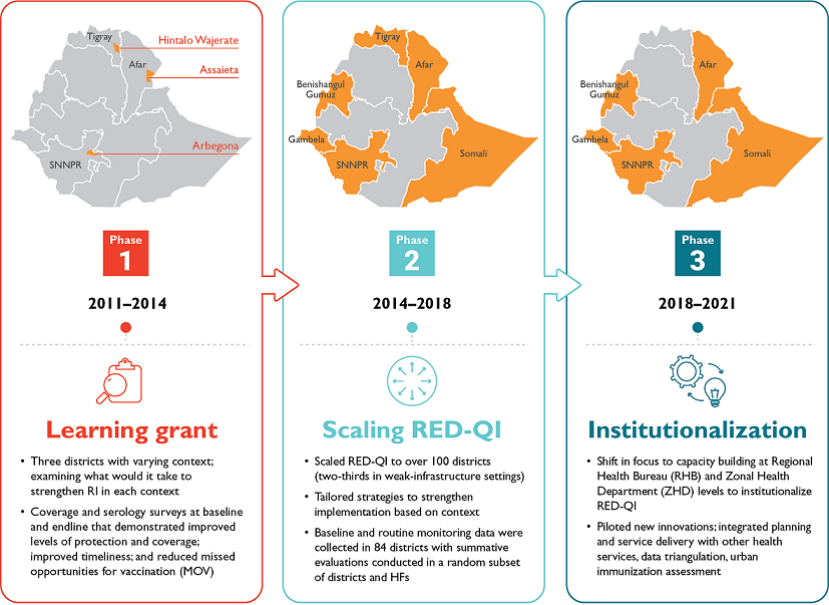
timeline for UI-FHS program implementation from 2011-2021

**Project scope:** JSI implemented the Universal Immunization through Improving Family Health Services (UI-FHS) project between 2011 and 2021 in six of 11 regions in Ethiopia, reaching over 2,700 health facilities in 103 districts. Participating districts were selected in collaboration with the Ethiopian MoH with each having at least one of the following characteristics: low immunization coverage (below 50 percent), a prevalence of measles and history of outbreaks, or categorization as either rural remote or with large nomadic populations. RED-QI implementation was initiated in three districts in 2011 and was expanded to an additional 100 districts in 2014. Figure 1 provides an illustration of the three phases of the UI-FHS project. The RED-QI approach was introduced through a three-step process with technical support provided over either 13 or 24 months, depending on the strength of the district´s health system.

**Key components of RED-QI:** Table 1 below outlines some of the key components and innovations of QI that were integrated into the RED strategy in Ethiopia.

**Table 1:** examples of activities that were implemented through the RED-QI approach to strengthen the RI system

RED strategy components	RED-QI components
**Planning and management of resources (including microplanning)**	- Sub-district (e.g., health facility level) EPI microplans - Engagement of communities in microplanning: mapping the location of populations (e.g., identifying hard to reach populations, travel routes for nomadic populations, refugee areas, etc.) and determining where and when services should be provided - Introduction of root cause and fishbone analyses and the “five whys” to identify the underlying causes of problems and explore barriers to service delivery - Plan-Do-Study-Act (PDSA) cycles to test solutions crafted by health workers and community members
**Engaging with communities**	- Quality Improvement Teams (QITs) of health workers and community members that focus on immunization and conduct PDSA cycles, trace defaulters, and obtain community input on planning, including optimal location and time for vaccination outreach sessions and problem-solving - Involvement of civil administrators to elevate issues and mobilize local resources - Engagement of non-health stakeholders in monthly meetings to review immunization data and respond to funding gaps
**Conducting supportive supervision**	- Increased focus on building the capacity of health workers and on-site mentorship - Revision of existing supportive supervision tools
**Monitoring and using data for action**	- Data quality self-assessment and improvement in data consistency across standard EPI reporting tools - Increasing health worker capacity to monitor coverage and dropout rates to inform planning **-** Quarterly Review Meetings with health personnel and local non-health stakeholders to review performance, encourage participants to solve problems by “thinking outside the box,” mobilize local resources, and flag problems that require national-level attention - Examination of coverage and dropout rates to determine health facilities most in need of supportive supervision - Focus on improving the quality of data collected at health facilities
**Reaching all eligible populations**	- Participatory community mapping to identify catchment populations - Use of QITs to obtain community input on optimal locations and times for vaccination sessions - Provision of technical support to plan and implement mobile and outreach services - Tracking of planned and conducted immunization sessions to monitor whether target populations are being reached

## Methods

This paper draws on quantitative and qualitative data from baseline (2014) and endline (2018) studies, program-monitoring studies, and two qualitative studies. Quantitative data were collected during baseline, endline, and project monitoring. Baseline data collection was a census of almost all health facilities in the six project regions. Endline data was collected at random from 110 health facilities in 18 project districts across the six regions; for each district we randomly selected 50% of the health centers (HCs) and 5-8 health posts (HPs) depending on the number of HPs in the district. All HFs included in the endline assessment were part of the baseline data collection for comparative purposes. The assessments were conducted using a structured questionnaire tailored to each level, which included information on human resources, cold chain and logistics, the functionality of HFs, and supportive supervision. Program monitoring data was collected on a rolling basis during implementation. The main source was supportive supervision visits conducted by JSI and MoH staff, who used an immunization-specific checklist.

In 2017, the project conducted a qualitative study to examine implementation of Plan-Do-Study-Act (PDSA) cycles to improve immunization service delivery. Data collection included 27 key informant interviews (KIIs) with district staff, HF staff, and project staff and 12 focus group discussions (FGDs) with QIT members at the district, facility, and community levels. Also in 2017, UI-FHS conducted a qualitative mid-program review to examine the rollout of RED-QI in the Afar and Somali regions of Ethiopia. The study examined contextual factors that influenced implementation and factors that hindered or facilitated sustainability. Data collection included 36 KIIs with regional, district, and sub-district HF staff and 11 FGDs with QITs at the district and community levels. Prior to all KIIs and FGDs, verbal and written consent was sought from all participants. Data was saved in secure folders; access was restricted to the research team. Findings were anonymized for all reports and publications so no response could be traced back to individuals. In Ethiopia, Institutional Review Board (IRB) approval for the “Use of Plan-Do-Study-Act Cycles for Strengthening Routine Immunization” study was obtained through the Ethiopian Public Health Institute´s Scientific and Ethical Review Committee under project number SERO-030-12-2016. The Mid-Program Review was determined exempt by the JSI IRB (OHRP IRB00009069 John Snow, Inc.). Baseline, endline, and monitoring data were part of the project´s routine data collection. The project sought and received verbal approval to collect these data from the MoH, district health offices, and HFs.

**Data analysis:** quantitative data were collected using hard copy files and were entered into Excel and transferred to Stata (version 14) for analysis. Baseline implementation data were compared to data from supervision visits using Microsoft Excel to track trends for key indicators. Due to the large number of HFs in districts and the varying degrees of supportive supervision received by facilities, only those with three or more supervision visits were included in the analyses presented in this paper. Findings from qualitative data collection were compiled in the form of KII and FGD transcripts. All data were coded in NVivo (Version 12), and findings were extracted based on predefined codes, using thematic content analysis techniques.

## Results

The RED-QI approach aimed to strengthen the Ethiopian health system to deliver higher quality and more equitable health services. The RED-QI intervention sought to build immunization managers´ and service providers´ capacity to plan, implement, and monitor equitable immunization services using QI tools and data for local problem-solving.

### Redesigning the planning process to engage communities and strengthen service delivery

Immunization microplanning is critical to identifying and targeting populations. At the start of the UI-FHS project, microplanning in HFs (health center and health post levels) in the project areas was very low. Before receiving UI-FHS support, only 12 out of 84 project districts (14 percent) and 9 out of 196 project HFs (5 percent) had a complete microplan. JSI provided district and HF staff with comprehensive training and support to build skills in developing and using immunization microplans. The RED-QI approach to microplanning is driven by bottom-up planning with community members to ensure that community needs inform immunization services. The microplanning training that UI-FHS provided included QI tools to improve the planning process, engagement of community members to identify and support community mapping, and post-training follow-up visits to support HFs in microplan implementation. Following the training and support, (75 out of 84) or 89 percent of participating districts and (173 out of 196) or 88 percent of participating HFs reported completing microplans.

*“We used to go out without proper planning for outreach and carrying the cold box, we used to wander around looking for target children which also affects the quality of the vaccine since the cold box gets warm. Now, we tell the QIT (Quality Improvement Team) about the outreach (two days before), and we are working in well-organized manner.” - Health Worker, health facility level*.

A key element of the microplanning process is participatory mapping, where community members and health workers map the location of communities and determine how to reach them with immunization services. Personnel from multiple health system levels said that they most appreciated two concepts from RED-QI: participatory mapping of catchment populations and using this information to determine where to provide outreach and mobile services. HF staff sometimes have limited knowledge of the locations of communities [24], especially nomadic communities, so engaging community members is essential to designing an effective catchment map and planning services. Community clan leaders were included in the mapping process to identify small communities, nomadic travel routes, and the best times and locations to reach these communities. Community engagement in catchment area mapping increased from baseline to endline in data collected from 18 districts and 110 HFs, 33% for district level and 26% for HF level. Although the microplanning process was successful, it required a great deal of time, training, follow-up, and supportive supervision. Health personnel said they found the microplanning tools complex, citing both their length and technical concepts. This made it challenging for HWs to update microplans regularly, with staff requiring ongoing support from supervisors. Health personnel noted that the RED-QI concepts introduced during training should be reinforced through on-the-job training and supportive supervision.

### Improvements in the management and delivery of immunization services

**Figure 2:**
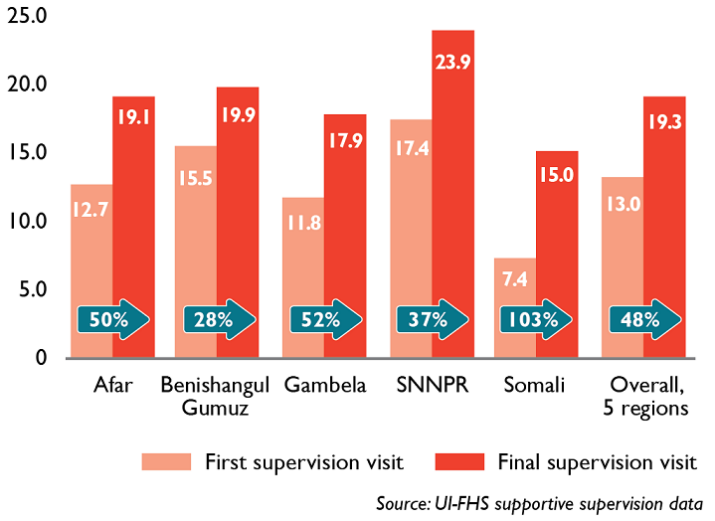
the regional average of total scores and percent change on EPI-specific supervision checklist, district level Ethiopia

Immunization staff were provided training supplemented by on-the-job mentoring, largely provided through supportive supervision visits. During supportive supervision visits, supervisors talked with staff about what was working well and the challenges they experienced. The visits also provided an opportunity to examine changes in HF performance over time. JSI was able to use changes over time in EPI-specific supervision checklist scores as a measure of RI system improvement at the district level (Figure 2, data in this figure is representative of districts that received three or more supervision visits, n=84. Regions include Afar, Benishangul-Gumuz, Gambela, SNNPR, and Somali. An aggregate score representing the 84 districts from 5 regions is presented at the far right). Summary 2016-2018 data from a standard EPI-specific checklist used in five regions where RED-QI was introduced shows that, on average, districts improved their checklist scores by 49 percent. These improvements suggest that the project´s capacity-building strategies improved EPI process indicators and strengthened the immunization system.

Despite improvement in overall HF scores, the PDSA study found that capacity to implement RED-QI methods varied by staff level. District staff had greater familiarity with RED-QI methods and could describe microplanning and mapping, PDSA cycles, and QITs, whereas frontline health workers were relatively less familiar with these RED-QI concepts. In a significant number of HPs, the project did not introduce PDSA cycles due to infrastructure and staffing weaknesses that would have rendered the concept difficult to implement. In facilities where PDSAs were used, respondents reported focusing more on the Plan, Do, and Act phases of the cycle than on the Study step. They reported finding great value in identifying root causes and testing new change ideas to address service problems but considered the Study step of analyzing and recording data difficult; this was especially true in HFs.

*“PDSA helped not only for immunization but also we used it for pregnant mothers´ follow-up and latrine construction for HHs and its use. It helps to identify the problems and their causes...” - Health Worker, health facility level*.

As part of the microplanning process, district staff and service providers designed static, outreach, and mobile immunization services to reach all communities, prioritizing those with high numbers of unvaccinated (i.e., zero-dose children) and underimmunized children. A comparison of baseline and endline data showed a 24 percent increase in HF use of defaulter tracking mechanisms to identify and bring underimmunized children to HFs for vaccinations.

### Increased capability to use data for action

JSI introduced activities to help health personnel and community members understand how the data available to them could be transformed into meaningful, useful information. JSI enhanced the capacity of immunization staff at all levels of the system to record and report accurate data and use data to improve programming. At the district level, JSI introduced a tailored version of WHO´s RED Categorization tool, which helped staff to analyze the performance of individual HFs based on immunization coverage data and solve problems related to increasing coverage through collaborative discussion and root cause analyses. Use of the RED Categorization tool increased from 51 percent at baseline (43 out of 84) to 79 (66 out of 84) percent at endline.

**Figure 3:**
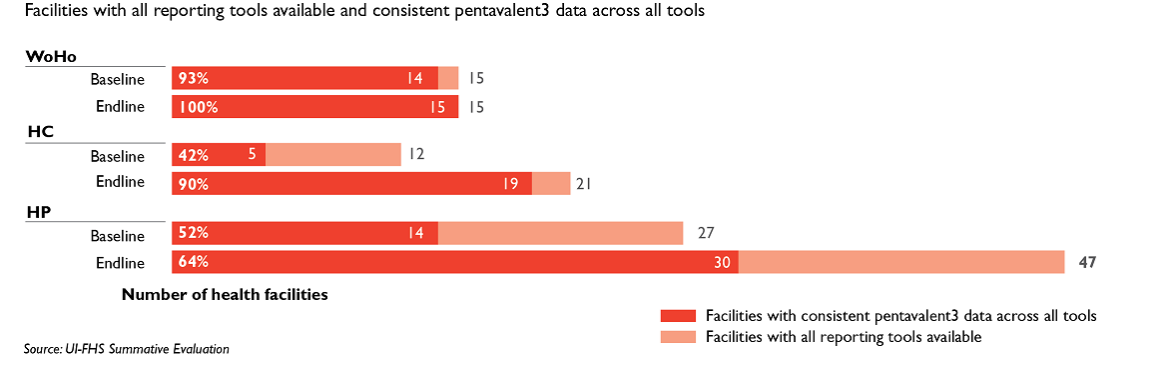
baseline and endline scores from facilities which have all reporting tools available and consistent pentavalent 3 data across all tools

JSI also urged immunization managers and service providers to use data to examine if (and how) their programming reached equity goals. JSI supported immunization staff to use their microplans to identify target populations and track whether they had been reached with immunization services. At the district level, tracking of service provision increased from 10 percent at baseline (8 out of 84) to 79 percent at endline (66 out of 84); in health centers, tracking increased from 8 percent (8 out of 97) to 88 percent (85 out of 97). This meant that district and HF staff were able to track if they were reaching more children. Using data from a sample of 35 HFs, the project found that 91 percent of planned static services and 80 percent of outreach services were conducted by endline, a considerable improvement from baseline when only 9 HFs (5 percent) had developed a microplan. JSI also worked to engender a sense of accountability and ownership of data, especially at the HF level. When data becomes something that health personnel use daily, rather than something they simply report up the chain, the quality of data recording and reporting typically improves. From baseline to endline, the project saw a 59 percent increase (from 39 to 62 percent) in the number of HFs reporting concordant data across three major immunization reporting tools (i.e., EPI register/family folders, monitoring charts, and monthly reports), indicating improved data accountability and quality (Figure 3).

With the introduction of RED-QI, QITs became a forum for identifying and addressing immunization service delivery problems and other health challenges. At the district level, QITs require collaboration between the EPI manager, health and supply chain managers, and finance directors; at the HF level, the community and the health workers must work together. From baseline to endline, the number of facilities with a QIT increased from 11 to 72 percent at the district level (n=18), from 24 to 85 percent at the health center level (n=37), and 21 to 73 percent at the HP level (n=73). Despite limited understanding of the PDSA cycle, HF QITs introduced process improvements to improve service delivery and carried out change ideas, such as locating unimmunized children. Where QITs consisted of health workers and community members, health workers reported viewing community members as key stakeholders who helped identify RI problems and suggest and implement solutions.

*“They [QIT] mobilize the community during EPI, identify and solve problems in the community, and support [health post] activities in the community.” - Health Worker, health facility level*.

The number of QITs at the health facility level with evidence of using data to improve the RI system increased from 28 percent (24 out of 87) at baseline to 55 percent (82 out of 150) at the final supervision visit. This suggests that health workers at all health system levels used local data to solve local problems.

### Challenges to RED-QI adoption

Despite improvements in process measures and vaccination coverage [20], program assessments revealed challenges with scaling and sustaining all components of the RED-QI approach. The majority of challenges were rooted in the health system (e.g., insufficiencies in human resources, funding for supportive supervision, and distribution of critical tools like monitoring charts). Staff turnover was a key impediment to the uptake and continuation of RED-QI practices, with limited or no transfer of knowledge and skills from those who had received training to new staff who had not. Understaffing and staff with insufficient time for RED-QI activities were additional key factors. This was especially true of more complex activities like PDSA cycles and QITs. Health managers and health workers reported widespread low motivation, limited commitment to quality, and poor linkages across government structures.

*“Due to absence of trained staff and high workload, I could not use PDSA. We believe on the continuity of the PDSA but due to shortage of staffs and high workload we can´t use PDSA for any service.” - Health Manager, district level*.

## Discussion

Program assessments suggest that RED-QI has helped strengthen several key aspects of the RI system including improvements in process measures (management and service delivery) and vaccination coverage [23]. The assessments also confirm existing evidence that QI approaches delivered through a comprehensive capacity-building strategy can strengthen district-level leadership and management [13,25]. The assessments suggest that the RED-QI approach strengthened the capacity of health workers to plan RI services, solve problems, improve program implementation, and enhance the quality and use of immunization data. RED-QI also encouraged partnerships between the health system and communities and engaged critical non-health stakeholders (e.g., local civil authorities, politicians, community leaders), who increased their support of immunization activities [21,26].

The project found microplanning the gateway activity to increasing equitable programming. The community-driven microplanning process generated more accurate, realistic plans, increased health worker ownership of, and follow-through on plans. Equally important, the process enabled health personnel and community members to understand and use data about the community. Because the RED-QI approach to microplanning incorporates QI tools like root cause analysis, problem prioritization, and problem-solving, participants described it as an effective means for creating plans that reach all communities and for enhancing health workers´ problem-solving capacities. Health personnel also used microplans to track progress, understand whether plans were implemented as designed, and assess whether HFs were reaching all communities, with a special emphasis on the un- and underimmunized. This study extended existing evidence on using QI tools to strengthen HWs´ skills to solve problems and design solutions to local problems. Additionally, the RED-QI approach was found applicable in a range of settings, with improvements observed in districts with strong health systems and those with weak health infrastructure and significant nomadic populations. Investing in building the capacity of health workers to solve local problems-especially at district and HF levels, produces tangible results. These findings are of particular importance as the global immunization community looks for innovations to reach zero dose children; investing in health worker capacity to identify and solve localized problems will be important to achieving IA2030 goals.

While adaptable to different settings, the RED-QI approach is a complex intervention requiring an ongoing commitment to training and on-the-job support. The RED-QI model was not fully implemented in all sites. Partial implementation was attributed to staff turnover, lack of ongoing reinforcement of key concepts, the complexity of certain aspects of the approach, and low staff motivation. These are similar to the findings of Stover *et al*. [25]. Health systems issues beyond the control of the district health team (e.g., insufficient human resources, limited funding for outreach and mobile services, periodic vaccine stockouts) affected the implementation of RED-QI at HF and health district levels.

Despite these challenges, applying QI tools to the RED strategy and providing technical assistance improved the capacities of immunization staff and enhanced the overall functionality of the RI system. We suggest that African countries examine whether (and how) the RED strategy is being implemented and consider using QI tools and processes to strengthen lagging components [27]. In particular, we recommend that countries invest in bottom-up microplanning using QI tools and support QITs to enhance community engagement in collaborative problem-solving.

The COVID-19 pandemic has accentuated the need for innovative approaches to strengthening health workers´ capacity and the systems in which they work. Elements of the RED-QI strategy could be essential to understanding how RI has been affected by the pandemic (e.g., using the RED Categorization tool to determine whether immunization coverage decreased during the pandemic as well as QITs and root cause analyses to understand why a drop occurred). RED-QI tools and methods could also help countries strengthen the equity and reach of their RI system and address challenges associated with COVID-19 vaccination.

### Limitations

Because the assessments from which this paper draws had varied timelines and were not designed as controlled studies in a closed environment, they were subject to many external, fluctuating variables. The assessment methodologies were designed to answer specific programmatic questions rather than test a common hypothesis, and thus did not employ a shared methodology, limiting the ability to conduct a rigorous comparative analysis across all parameters. The low and fluctuating quality of administrative vaccination coverage data impedes the ability to track the relationship between RED-QI inputs and immunization coverage. In fact, some RED-QI activities designed to improve data quality were expected to result in lower coverage rates. This contributed to the need to identify and track alternative indicators of immunization system performance, as described in this paper; the relationship between these indicators and RED-QI can be established with reasonable certainty.

## Conclusion

There is strong evidence to suggest that the RED-QI approach strengthens the capacity of health personnel to implement the essential elements of a RI system. The RED-QI approach increased health workers´ capacity to define HF catchment areas, identify and target communities for immunization services, and develop robust immunization microplans to reach those populations. Improved service delivery planning (e.g., resource mobilization for additional immunization sessions, reaching communities where they live) expanded the reach of immunization services, thereby improving service equity. These results are consistent with those found in the Manyazewal study conducted in Ethiopia [13]. These findings suggest that the RED-QI approach could be adapted and successfully implemented in other EPI across Africa.
